# Metagenomics Reveals Microbial Community Shifts Associated With Contrasting Anthropogenic Impacts in Freshwater Sources of A Coastal Protected Area in Southeastern Brazil

**DOI:** 10.1002/wer.70567

**Published:** 2026-09-07

**Authors:** Bruna Barbosa de Paula, Marize Pereira Miagostovich, Camille Ferreira Mannarino, André Vinicius Costa Ribeiro, Natália Maria Lanzarini, Carla Santos de Oliveira, Shênia Patrícia Corrêa Novo

**Affiliations:** ^1^ Laboratory of Comparative and Environmental Virology Oswaldo Cruz Institute, Oswaldo Cruz Foundation Rio de Janeiro RJ Brazil; ^2^ Post‐Graduate Program in Public Health and Environment, National School of Public Health Sergio Arouca Oswaldo Cruz Foundation Rio de Janeiro RJ Brazil; ^3^ Department of Sanitation and Environmental Health, National School of Public Health Sergio Arouca Oswaldo Cruz Foundation Rio de Janeiro RJ Brazil; ^4^ Laboratory of Arboviruses and Hemorrhagic Viruses Oswaldo Cruz Institute, Oswaldo Cruz Foundation Rio de Janeiro RJ Brazil

**Keywords:** antimicrobial resistance, bioindicators, conservation units, environmental monitoring, water quality

## Abstract

This study aimed to characterize freshwater microbial communities, environmental drivers, and anthropogenic impact patterns across three sites on Marambaia Island (southeastern Brazil) using metagenomics. Samples collected from freshwater sources used for human consumption were processed through concentration, nucleic acid extraction, and sequencing on the Illumina NextSeq 2000 platform. A total of 67.2 million reads were assembled into 89,230 bacterial contigs, mostly attributed to Gammaproteobacteria, Alphaproteobacteria, and Betaproteobacteria. Sites under lower anthropogenic influence exhibited higher microbial diversity, whereas impacted sites showed enrichment of opportunistic and fecal‐associated genera. A heterogeneous anthropogenic impact profile was observed across sites, corroborated by the proposed Anthropogenic Impact Index (AII). Fourteen antimicrobial resistance genes conferring resistance to *beta*‐lactams, quinolones, sulfonamides, tetracyclines, and macrolides were detected predominantly in sewage‐impacted areas, indicating potential diffuse contamination. Redundancy analysis revealed that environmental variables explained 88.1% of microbial community variation, with conductivity, salinity, and turbidity as key drivers. These findings demonstrate the applicability of metagenomics as a powerful tool for assessing microbial diversity, ecological dynamics, and contamination risks in vulnerable freshwater systems.

## Summary

- First shotgun metagenomic assessment of freshwater sources within a Brazilian coastal protected area inhabited by a traditional community.
- Contrasting anthropogenic pressures were associated with distinct freshwater microbial community signatures.
- Antimicrobial resistance genes reinforced evidence of human‐associated microbial contamination.
- Human‐associated bacterial genera supported the Anthropogenic Impact Index (AII).
- Environmental variables explained microbial community variation through redundancy analysis (RDA).

## Graphical abstract

Metagenomics revealed distinct anthropogenic signatures in the microbiome of freshwater sources on Marambaia Island, supported by the Anthropogenic Impact Index (AII). Impacted sites showed lower microbial diversity, enrichment of fecal‐associated taxa, and 14 antimicrobial resistance genes, whereas conductivity, salinity, and turbidity explained 88.1% of bacterial community variation.

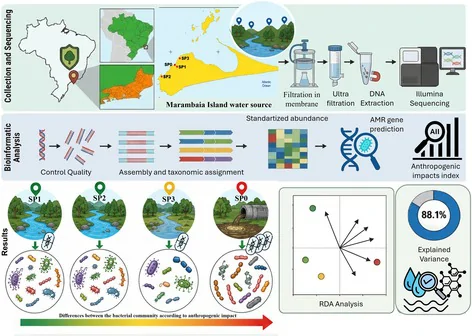

## 1 Introduction

Aquatic matrices represent complex environments, as they contain a wide variety of coexisting microorganisms and play significant roles in biogeochemical cycles. Distinct aquatic matrices exhibit microbial signatures shaped by their physicochemical characteristics, nutrient availability, and environmental pressures, resulting in marked differences in microbial community composition across freshwater, marine, and other water systems (Zhang et al. 2023).

Freshwater microbial communities contribute to fundamental ecological processes, such as nutrient cycling, organic matter degradation, biofilm formation, and bioactive substance production and purification (Battin et al. 2023). Therefore, understanding the role of microorganisms in regulating water quality and maintaining aquatic ecosystem health is essential to human well‐being, including food and water security (Zinger et al. 2012).

Disturbances in these communities, particularly those driven by anthropogenic pressures, can lead to reduced biodiversity, functional imbalance, and impaired biogeochemical cycling, compromising water quality, ecosystem resilience, and the provision of essential ecosystem services (Behera et al. 2023). This vulnerability is particularly consequential in protected areas, where freshwater sources often serve dual roles as ecological reservoirs and as drinking or domestic water supplies for resident communities, placing conservation objectives and human water security needs in close proximity.

Advances in high‐throughput sequencing have transformed environmental microbiology by enabling direct, comprehensive profiling of aquatic microbiomes. Metagenomics surpasses conventional microbial water quality monitoring by revealing a broader spectrum of microorganisms, including unculturable taxa, opportunistic pathogens, and functional genes (Bibby et al. 2019; Rout et al. 2025). Brumfield et al. (2020) demonstrated that metagenomic surveillance can detect opportunistic pathogens, virulence factors, and antimicrobial resistance (AMR) genes even in water samples where conventional fecal indicators are absent. Beyond pathogen detection, this approach allows for a holistic assessment of microbial dynamics and latent environmental risks (Gregory et al. 2019; Behera et al. 2021).

Despite these advancements, freshwater microbiomes in protected areas inhabited by traditional populations remain understudied. These zones represent fragile socio‐environmental interfaces where precarious sanitation and anthropogenic pressures directly threaten the water consumed daily by local populations, even under formal environmental protection status.

This study aims to assess patterns of anthropogenic impact across freshwater sources within the Sustainable Use Conservation Unit in Mangaratiba, Rio de Janeiro, by characterizing microbial communities, exploring environmental drivers, and detecting AMR genes. Additionally, we propose an Anthropogenic Impact Index (AII) to quantitatively evaluate human influence on these ecosystems, providing a robust framework to support conservation and management strategies in protected areas.

## 2 Materials and Methods

### 2.1 Study Area and Sampling

This study was conducted on Marambaia Island, a Coastal Sustainable Use Conservation Unit located in the municipality of Mangaratiba, Rio de Janeiro, Brazil (23°03′ S, 43°36′ W). The area comprises extensive Atlantic Forest, including coastal vegetation and mangroves, and is characterized by a high level of environmental conservation. The island is inhabited by a traditional community whose primary economic activity is fishing, with limited access to basic sanitation infrastructure and reliance on untreated freshwater sources for drinking and domestic use.

Three main populated areas were defined for this study: the central region (SP1), which concentrates the unique health, educational, and commercial facilities, and two peripheral regions (SP2 and SP3), characterized by lower population density and greater preservation of native vegetation (de Paula et al. 2024).

To account for environmental fluctuations and temporal dynamics, this study incorporated physicochemical data on water quality obtained from a comprehensive one‐year monitoring program, previously established by de Paula et al. (2024). Utilizing a multiseasonal dataset provides a robust environmental baseline that far outweighs the limitations of a single momentary snapshot, ensuring that correlations between environmental variables and microbial community structures reflect ecological patterns rather than short‐term transient variations. These included conductivity, salinity, turbidity, pH, and dissolved oxygen. A summary of these parameters is provided in Appendix A (Table A1).

**FIGURE 1:**
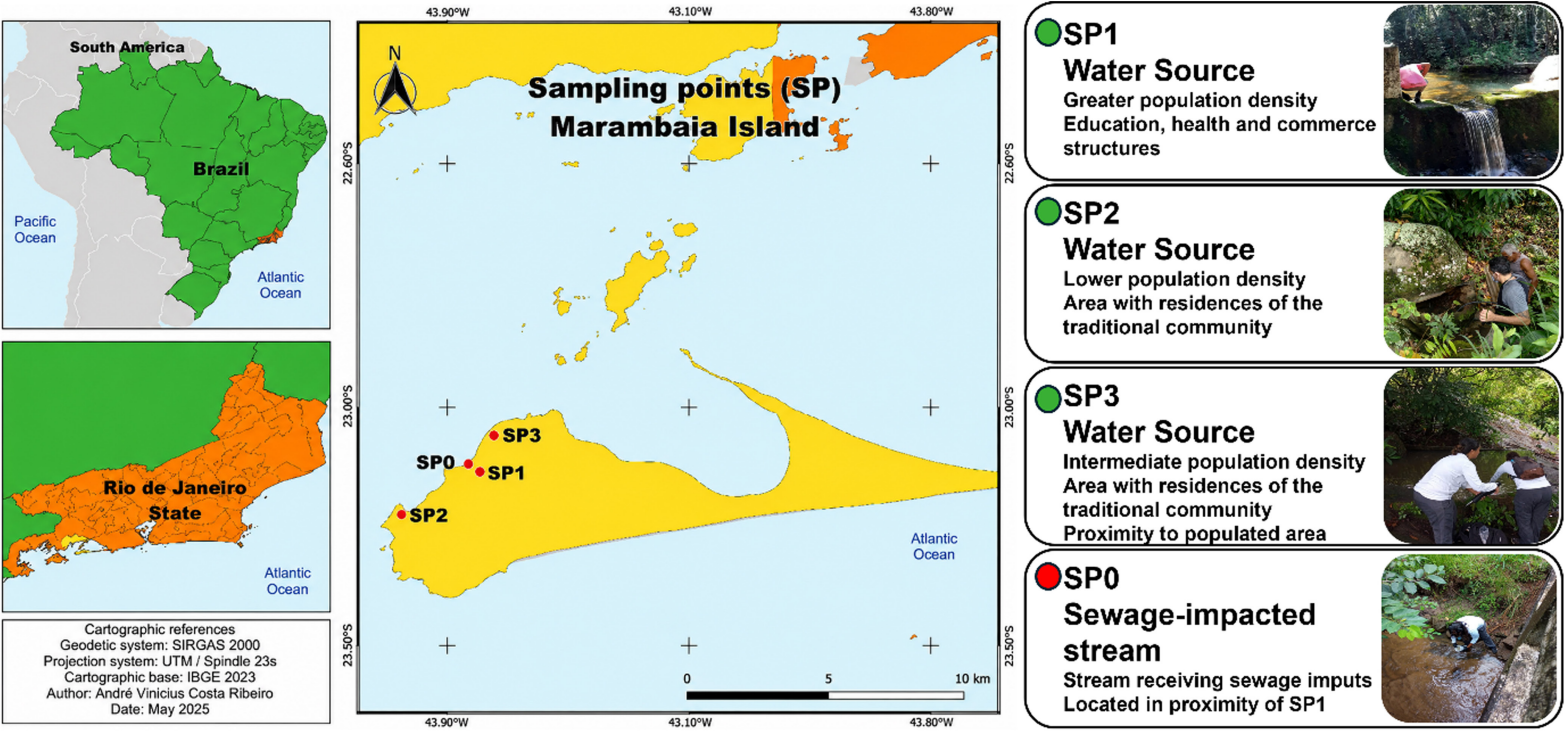
Location and characteristics of freshwater sampling sites on Marambaia Island, Rio de Janeiro State, southeastern Brazil. SP0: sewage‐impacted water (central area); SP1: freshwater source (central area); SP2: freshwater source (peripheral area south of central area); SP3: freshwater source (peripheral area north of central area). Coordinates are referenced to the SIRGAS 2000 geodetic system (UTM projection).

Based on these prior findings, freshwater samples were collected in February 2023 for metagenomic analysis. In addition to the previously defined sites (SP1–SP3), an additional sampling point (SP0) was included (Figure 1), representing a location with continuous input of untreated domestic sewage and serving as a positive control for anthropogenic impact.

At each site, three independent water samples were collected once at different times of the day (morning, afternoon, and evening) and subsequently pooled to generate a composite sample representing the daily microbiological profile of each location. Samples were collected in 2 L polypropylene bottles, stored on ice, and transported to the Laboratory of Comparative and Environmental Virology at the Oswaldo Cruz Institute (Rio de Janeiro, Brazil), where concentration, nucleic acid extraction, and sequencing preparation were performed. Prior to processing, samples were maintained at 4°C, and the concentration step was conducted within 24 h of collection.

### 2.2 Sample Concentration, Nucleic Acid Extraction, and Next‐Generation Sequencing

Sample concentration was performed employing a modified version of the electronegative membrane filtration method described by Katayama et al. (2002). The samples were pre‐treated with 5 mL of a 2 M MgCl_2_ solution acidified to pH 3.5 using 200 μL of a 6 N HCl solution and subsequently filtered through cellulose membranes (142 mm × 0.45 μm, Millipore) using a pressure system (Millipore), followed by membrane rinsing with 350 mL of 0.5 mM H_2_SO₄ (pH = 3.0). For neutralization and elution, the membranes were immersed in Petri dishes containing 15 mL of a 3 mM NaOH solution (pH 10.5–10.8) under mechanical agitation for 10 min in the orbital shaker to contribute to the elution of the material trapped in the membrane.

The eluates were supplemented with 50 μL of TEX 100X buffer and 50 μL of 50 mM H_2_SO₄ and transferred to an Amicon (Merck, Darmstadt, Germany) Centrifugal Filter Unit 50 KDa for ultrafiltration (1400 ×*g* for 7 min). The concentrated samples were then transferred to 2 mL microtubes and stored at −20°C until nucleic acid extraction, when samples from the same site were pooled.

In the next step, the nucleic acid extraction was performed using a Biopur Nucleomag Virus kit (Mobius Life Science, Brazil) without the addition of an RNA carrier and quantified using a Qubit 4.0 fluorimeter (Invitrogen Thermo Fisher Scientific, Waltham, Massachusetts, USA) according to the manufacturer's instructions. Reverse transcription was carried out followed by a polymerase chain reaction for RNA virus amplification. To ensure sample purity and remove contaminating free nucleic acids, purification was performed with an AMPure XP beads kit (Beckman Coulter, Indianapolis, Indiana, USA). The purified samples were then quantified using the same Qubit 4.0 fluorimeter. A blank control (DNase/RNase‐free water) was included in all steps, and DNA amplification was verified by agarose gel electrophoresis according to Hong et al. (2020).

For shotgun metagenomic sequencing, DNA libraries were prepared using a Nextera XT DNA Library Preparation Kit (Illumina, San Diego, California, USA). Library size distribution was assessed using a 2100 Bioanalyzer (Agilent, Santa Clara, California, USA). The shotgun paired‐end sequencing approach (2 × 150 bp) was applied employing an Illumina NextSeq 2000 Sequencer at the Oswaldo Cruz Foundation genomic facility.

### 2.3 Bioinformatics and Statistical Analyses

Raw sequences generated by the NextSeq 2000 platform (Illumina, USA) were converted to paired‐end FASTQ files. Quality control was assessed before and after trimming using FastQC (Andrews 2010). Reads were trimmed using Fastp v0.24.1 (Chen et al. 2018) with a Phred quality score threshold of 30. Trimmed reads were then mapped against the human reference genome (GRCh38) using Bowtie2 v1.0.1 (Langmead and Salzberg 2012) to remove host‐derived sequences and potential contaminants.

A k‐mer‐based taxonomic classification was performed on the filtered reads using Kraken2 v2.1.3 (Wood et al. 2019) with the pre‐built RefSeq Plus PF database (including protozoa and fungi; downloaded on 2024‐07‐15), followed by abundance estimation using Bracken (Lu et al. 2017). Taxonomic data were standardized using Z‐score transformation to enable direct comparison of relative taxon enrichment across sampling sites, independent of absolute abundance scales considering the 50 most abundant taxa among the collection points.

Alpha diversity was assessed using the Shannon index (Shannon 1948) to evaluate intra‐sample diversity. Beta diversity was assessed using Bray–Curtis dissimilarity (Bray and Curtis 1957) to evaluate inter‐sample diversity and visualized through non‐metric multidimensional scaling (NMDS) performed using the metaMDS function from the vegan package (Oksanen et al. 2022) with two axes (k = 2), 100 iterations (trymax = 100), and three random starts to ensure convergence. Ordination quality was evaluated using the stress value.

Redundancy analysis (RDA) was performed using the rda() function from the vegan package (Oksanen et al. 2022) to identify patterns in microbial community composition associated with anthropogenic impacts. The following physicochemical parameters obtained from de Paula et al. (2024) were included as explanatory variables: dissolved oxygen, temperature, salinity, oxidation–reduction potential (ORP), and total dissolved solids (TDS).

An anthropogenic impact index (AII) designed to represent human‐associated microbial contamination, based on the occurrence of bacterial genera commonly associated with human fecal contamination and opportunistic or pathogenic microorganisms, being possible to quantify the degree of anthropogenic influence on microbial communities, calculated as follows:

$$\begin{array}{c} \mathrm{AII}=\text{ΣAbundance}\_\text{markers}/\\ \;\left(\text{ΣAbundance}\_\text{markers}+\text{ΣAbundance}\_\text{environmental}\right) \end{array}$$

where *markers* refers to genera associated with fecal contamination or human activity, and *environmental* refers to genera typically associated with natural aquatic environments. This index provides a relative measure of anthropogenic influence and was used to support ecological interpretations alongside multivariate analyses.

All metagenomic analyses were performed through the Galaxy Microbiome Web Platform (https://microbiology.usegalaxy.eu/, The Galaxy Community 2024). Statistical analyses were carried out using R v4.2.0 (R Core Team 2022) with RStudio v1.4.1717 (RStudio Inc. 2022) and Jamovi v2.3.0 (The Jamovi Project 2023).

### 2.4 Assembly, Gene Prediction and AMR Gene Annotation

The reads were assembled into contigs and scaffolds using MetaSPAdes (v.4.1.0) with default settings for paired‐end reads, k‐mer sizes of 21, 33, 55, and 77, and auto‐set Phred quality offsets (Nurk et al. 2017). Assembly quality was checked through the QUAST v.5.3.0 software.

AMR gene detection was carried out using Abricate (v1.0.1) to screen scaffolds against the NCBI Bacterial Antimicrobial Resistance Reference Gene Database and ResFinder Database. A minimum identity threshold of 60% was applied due to gene fragmentation (Seemann 2016). This threshold was adopted due to the intrinsic characteristics of environmental metagenomic datasets, which often consist of short and fragmented contigs as shown in previous studies (Bengtsson‐Palme et al. 2018). Taxonomic assignment of scaffolds containing AMR gene fragments was performed by comparing their sequences against the NCBI non‐redundant (nr) database using BLAST.

## 3 Results and Discussion

### 3.1 Sequencing Quality Metrics

A total of 67.2 million paired‐end reads were generated across the four sampling points (SP0–SP3) and the negative control (CTR‐). Duplication rates ranged from 7.7% to 17.4%, with higher rates observed in the negative control and SP1. Adapter contamination ranged from 0.8% to 5.9% was effectively removed during preprocessing. After quality filtering and adapter trimming, read counts were reduced by 37%–48%, a common outcome for environmental metagenomic datasets due to the removal of low‐quality and non‐target sequences.

The remaining reads were of high quality, with more than 90% of the bases exhibiting a *Phred score* above 30 (Q30). GC content ranged from 41.1% to 48.4%, consistent with diverse microbial communities and indicating the absence of strong compositional bias. Overall, sequencing metrics demonstrated high data quality and suitability for downstream taxonomic and functional analyses (Table 1).

**TABLE 1:** Aggregate results regarding the quality metrics of all water samples and the blank control.

Sample	Duplication rate	> Q30	Mb Q30 bases	Reads before trimming	Reads after trimming	GC content	PF	Adapter (%)	Mean length (bp)
CTR—	10.7%	92.7%	1505.6 Mb	11.7 M	11.2 M	46.2%	98.9%	2.2%	136
SP0	8.5%	90.8%	1721.4 Mb	14.1 M	8.71 M	43.7%	98.9%	5.9%	137
SP1	17.4%	93.7%	1796.8 Mb	16.1 M	8.45 M	48.4%	99.3%	0.8%	119
SP2	7.7%	92.7%	1586.6 Mb	12.3 M	7.82 M	43.9%	99.4%	1.5%	140
SP3	8.5%	92.2%	1675.4 Mb	13.2 M	8.02 M	41.1%	99.3%	2.7%	139

Sequencing depth and coverage varied across sampling sites. The highest average sequencing was observed in SP2 (530×), whereas SP0 showed the lowest depth (40×). Average genome coverage (%) followed a different pattern, ranging from 6.06 to 28.01. High‐quality sequencing data are essential to ensure the accuracy and reliability of taxonomic annotation.

### 3.2 Microbial Profiling and Community Analysis

**FIGURE 2:**
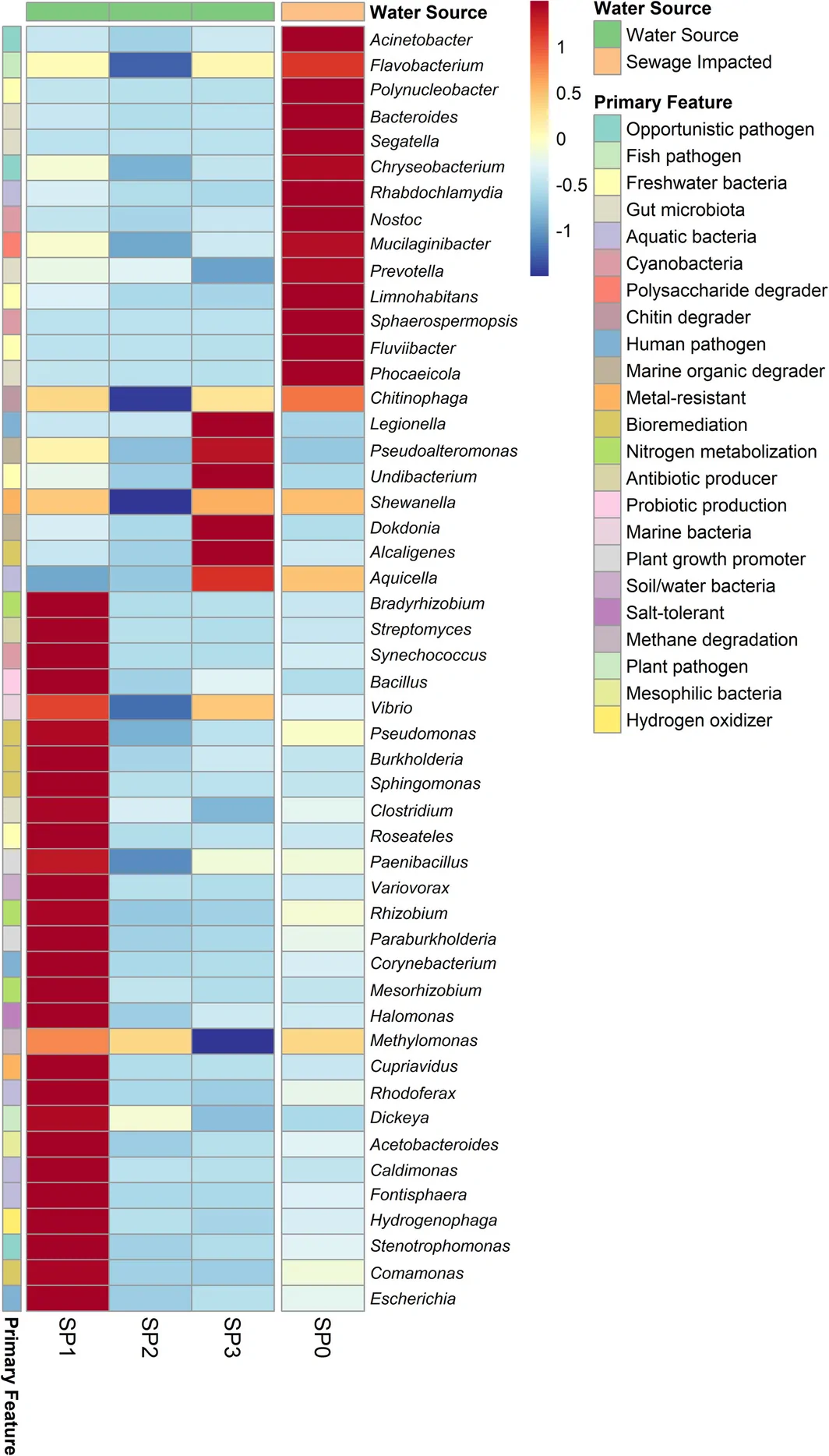
Standardized abundance of bacterial genera and their associated ecological niches at freshwater sources at Marambaia Island.

A total of 35.2 million bacterial paired‐end reads were generated and assembled into 89,630 contigs. Taxonomic profiles were generated based on genus‐level assignments. Analysis of microbial community composition revealed distinct profiles across the four sampling sites (Figure 2). These differences were primarily associated with site‐specific environmental conditions, including marine influence, biogeochemical activity, the presence of fecal contamination indicators, and varying degrees of anthropogenic impact, highlighting their role as key drivers of microbial diversity.

Proteobacteria was the dominant phylum across all samples, comprising representatives of Gammaproteobacteria, Alphaproteobacteria, and Betaproteobacteria. This group includes a wide range of genera associated with both environmental processes and human health and is commonly reported as dominant in freshwater systems, particularly in tropical regions (Köchling et al. 2017).

A heterogeneous bacterial community, characterized by the coexistence of environmentally beneficial and potentially pathogenic taxa, was observed in SP1. The community exhibited a high relative abundance of genera associated with soil and aquatic ecosystem functions, including nitrogen‐fixing and plant growth‐promoting symbionts (*Mesorhizobium*, *Bradyrhizobium*, *Rhizobium*), as well as taxa involved in methane oxidation and pollutant degradation (*Variovorax*, *Burkholderia*, *Alcaligenes*).

However, genera of public health relevance, including *Escherichia, Acinetobacter, Legionella, Salmonella, Helicobacter, Vibrio*, and *Pseudomonas*, were also detected at low relative abundances. These taxa are commonly associated with fecal contamination and opportunistic infections, indicating potential microbiological risks to human health (Ahmed et al. 2021; Almaghrabi et al. 2024).

In contrast, SP2 exhibited reduced diversity, with a predominance of sequences affiliated with genera such as *Legionella*, *Clostridium*, and *Prevotella*, which include species of public health relevance. Environmental taxa associated with ecosystem processes were also detected, including methane‐oxidizing bacteria (*Methylomonas*) and organic matter degraders (*Flavobacterium*). These findings indicate that, despite a lower anthropogenic impact, suggested by both the community composition and the site's greater distance from the urban center, there remains a critical concern regarding the circulation of pathogenic bacteria.

SP3 exhibited a high abundance of sequences assigned to *Legionella*, which represented one of the most dominant genera at this site. This observation suggests environmental conditions that may favor its persistence and proliferation, such as the presence of protozoan hosts (amoebae) and biofilm‐forming niches—well‐known ecological reservoirs for this genus. Although genus‐level taxonomic assignment cannot confirm the presence of pathogenic species or viable cells, the evidence of *Legionella* circulation raises important water quality considerations, particularly for freshwater systems used for human consumption. Furthermore, the detection of *Staphylococcus* in water source suggests human‐derived contamination, associated with non‐fecal sources, and may represent an additional risk in scenarios of recreational and domestic uses.

Despite the presence of pathogens, the results also show an abundance of marine and halotolerant bacterial genera, including *Pseudoalteromonas, Dokdonia, Shewanella*, and *Rheinheimera*, suggesting that this site is under marine influence from saline intrusion, aerosols, or tidal input.

The microbial profile of SP3, characterized by the co‐occurrence of genera associated with fecal sources (*Lactobacillus*), non‐fecal human contamination (*Staphylococcus*), and environmental opportunists (*Legionella*), builds upon and refines previous findings for this site (de Paula et al. 2024). Although the prior study, based on physicochemical and conventional microbiological indicators, flagged compromised water quality despite the site's non‐central location, the metagenomic data presented here provide a higher‐resolution diagnosis: the contamination pattern is diffuse in origin, arising from a combination of interacting fecal and non‐fecal sources. This multi‐source impact model better explains the site's complex microbial assemblage, distinguishing it from a point‐source pollution signature.

As a methodological reference for severe fecal contamination, SP0 (a site known to be directly impacted by untreated domestic sewage) was analyzed to validate the metagenomic inference framework applied to the source water sampling points (SP1–3). At this site, Proteobacteria, Bacteroidetes, and Firmicutes were dominant, with a representation of genera commonly detected in wastewater (*Escherichia, Shigella, Clostridium, Bacteroides*), confirming intense organic matter and sewage input. Furthermore, the detection of genera harboring opportunistic pathogens (*Acinetobacter, Legionella, Enterobacter, Pseudomonas, Moraxella*) and specific pathogens such as *Vibrio* underscores the significant human health risks associated with this degree of impact.

The microbial composition observed in this study is consistent with the emerging consensus that metagenomics‐derived microbial communities can serve as robust bioindicators for monitoring ecosystem health, toxicology, and human impact (Rout et al. 2025). This bioindicator potential is grounded in the well‐established dynamic by which natural waters influence, and are in turn influenced by, the metabolism of aquatic organisms (Libânio 2016).

The structure of the microbial community observed in this study bears a strong resemblance to the patterns described in tropical river systems of Ilha Grande Bay (Bacha et al. 2025), which is a neighboring region. The persistent dominance of *Proteobacteria* and the enrichment of *Enterobacteriaceae* (*Escherichia*, *Shigella*) in sites subjected to greater population density corroborate the hypothesis that contamination imposes a recurrent taxonomic signature in Rio de Janeiro watersheds, even in less impacted areas and reinforces that the freshwater microbiota is being shaped by homogeneous anthropogenic pressures, resulting in shared public health risks among different ecosystems in the region.

**FIGURE 3:**
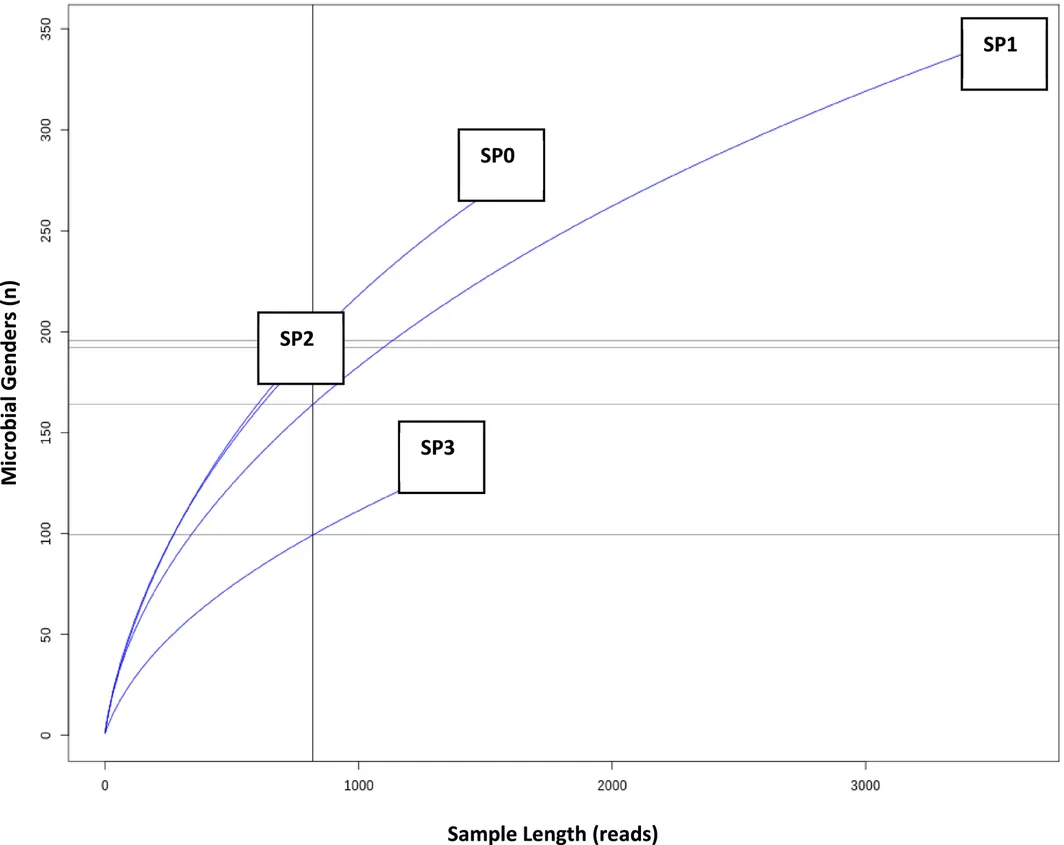
Associated rarefaction curve diversity of each sample.

Building directly upon the taxonomic shifts, alpha and beta diversity metrics quantified how structural changes impact community stability across the distinct sites. The rarefaction analysis indicated that sequencing depth was sufficient to capture most of the microbial diversity, with clear differences in species richness among sites (Figure 3). SP1 displayed the highest richness, as evidenced by the uppermost rarefaction curve, whereas SP3 showed the lowest. SP0 and SP2 exhibited intermediate curves, indicating moderate richness levels. Corroborating these findings, the Shannon diversity index—which accounts for both richness and evenness—revealed the highest intra‐sample diversity at SP1 (5.29), reflecting a taxonomically rich and evenly structured community. Intermediate diversity was observed at SP0 (4.45) and SP2 (4.27), whereas SP3 presented the lowest value (3.69).

**FIGURE 4:**
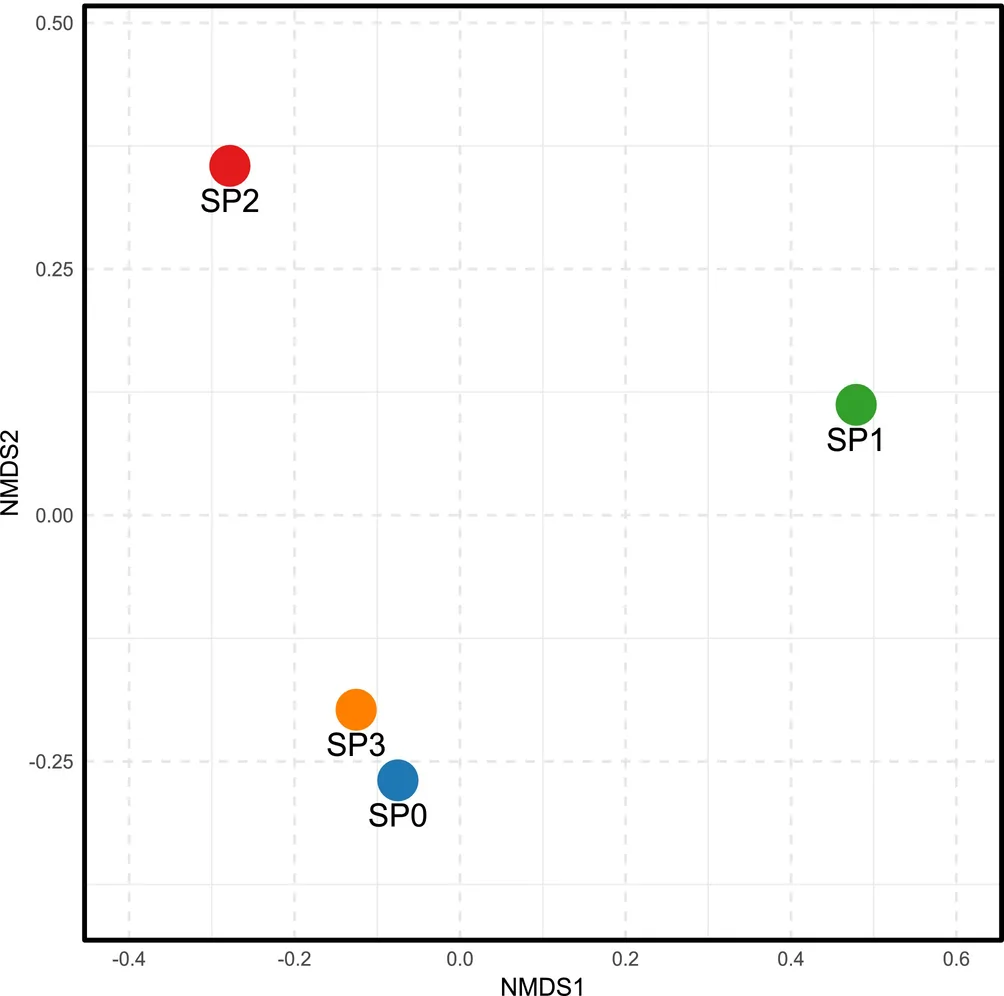
Non‐metric multidimensional scaling ordination of microbial community composition based on Bray–Curtis dissimilarity among sampling points (SP0–SP3) at Marambaia Island. Points represent individual samples, and distances between them reflect the degree of compositional dissimilarity (stress = 0.22).

According to Loreau et al. (2001), higher biodiversity enhances ecosystem resilience by maintaining functionality despite species loss and buffering against environmental stressors. This principle provides a framework for interpreting the beta diversity patterns observed in this study. The Bray–Curtis dissimilarity analysis, visualized through Non‐Metric Multidimensional Scaling (NMDS) with two axes (k = 2), 100 iterations (trymax = 100), and three random starts to ensure convergence revealed a clear spatial segregation of sampling sites driven by community composition (Figure 4).

The NMDS ordination placed SP1 and SP2 at opposite extremes along the first axis (NMDS1), consistent with the highest recorded dissimilarity value (0.795). This strong separation reflects the ecological contrast between a taxonomically rich, evenly structured community (SP1) and one dominated by a restricted set of genera, including potential pathogens (SP2). In contrast, SP0 and SP3 clustered more closely in the ordination space, exhibiting the lowest pairwise dissimilarity (0.586). This proximity suggests convergent shifts in community structure driven by distinct but overlapping stressors: raw sewage input at SP0 and a combination of diffuse fecal contamination, non‐fecal human impact, and saline intrusion at SP3.

The NMDS thus translates quantitative dissimilarity into a visual ordination space, wherein the distance between points reflects the magnitude of compositional change among the sites. Taken together, the congruence between alpha diversity metrics and beta diversity ordination delineates a spectrum of anthropogenic disturbance, demonstrating how microbial community structure mirrors both the intensity and the nature of environmental impact across the sites.

The NMDS ordination (stress = 0.22) captured the heterogenic composition among sites. Although the stress value falls within the upper acceptable range, attributable to the small number of sampling points and the constraints of two‐dimensional representation, the ordination pattern was fully consistent with the underlying Bray–Curtis dissimilarity matrix.

In accordance with fundamental ecological theory, high taxonomic evenness and richness buffer ecosystems against stress, explaining the structural stability observed at SP1 (Loreau et al. 2001). Conversely, the compression of diversity observed at SP3 reflects intense environmental filtering. This phenomenon aligns with observations in coastal wetland systems globally, where environmental stressors, specifically salinity gradients and anthropogenic encroachment, significantly restructure microbial communities by favoring only stress‐tolerant taxa at the expense of rare and sensitive species (Liu et al. 2023).

Our findings mirror this global trend, indicating that even in geographically isolated systems like Marambaia, the microbial assembly is governed by predictable ecological responses to environmental quality rather than stochastic variation. Furthermore, these diversity patterns are consistent with regional studies in tropical river basins. Köchling et al. (2017) documented similar declines in microbial richness in response to urban pollution in Brazilian tropical systems, confirming that anthropogenic impact acts as a primary diversity‐reducing driver across the region.

### 3.3 Assembly, Functional Analysis and Antibiotic Resistance Marker (AMR) Gene Detection

The taxonomic and diversity analyses revealed a clear segregation of sampling sites based on microbial community structure and identified the enrichment of potentially pathogenic genera in anthropogenically impacted points. To evaluate whether these compositional patterns are accompanied by differences in the AMR potential of the microbial communities, AMR genes were identified directly from the assembled shotgun metagenomic data and compared across sites.

De novo assembly using metaSPAdes generated a total of 417,419 contigs distributed across the four samples, of these, 89,230 are taxonomically classified as bacterial. Despite the heterogeneous number of contigs per site, N50 and N90 values were similar among samples, indicating comparable contig size distributions. GC content varied moderately across sites (40.22%–46.32%), consistent with the differences in bacterial community composition reported in the taxonomic analysis. The proportion of taxonomically assigned contigs ranged from 26.49% to 43.04% (Table 2).

**TABLE 2:** Assembly‐generated contig quality metrics per water sample from Marambaia Island.

	SP1	SP2	SP3	SP0
# contigs (≥ 0 bp)	42,965	95,012	186,458	92,984
# contigs (≥ 1000 bp)	1451	3732	2849	920
Total length (≥ 0 bp)	18,286,923	46,173,820	76,413,504	38,299,572
Total length (≥ 1000 bp)	25,738,94	69,044,74	44,923,96	15,261,03
# contigs	33,505	86,082	139,937	79,512
Largestcontig	15,424	45,687	20,391	9850
Total length	17,051,057	44,983,230	70,211,135	36,540,548
GC (%)	46.32	43.9	40.22	43.44
N50	455	473	485	436
N90	361	364	368	361
auN	801.5	1011.4	649.9	543.2
L50	11,506	29,246	52,909	31,991
L90	28,674	73,415	120,152	69,172
# *N*'s per 100 kbp	4.11	4.69	4.24	0.9
Taxonomy assigned (%)	41.07	40.61	26.59	43.04
Bacterial contigs	10,799	23,064	29,130	26,637

The scaffolds generated from the assembly contigs were used to predict putative proteins and genes through the FragGeneScan (Galaxy Version 1.30.0) tool, which is useful in detecting fragmented genes in short reads (Rho et al. 2010), using an Illumina sequencing read model with a 1% error rate. The scaffolds were screened employing two AMR genes databases: NCBI and Resfinder, relative to a minimum of 60% identity and 2% depth, as they comprise a short‐read metagenome, where fragmented reads can prevent a gene from being detected in its entirety.

The detection of antimicrobial resistance (AMR) determinants in short‐read metagenomic datasets presents inherent technical challenges, primarily driven by gene fragmentation and low sequencing depth for rare community members. To overcome this, the use of specialized predictors like FragGeneScan is essential for accurate protein and gene recovery from error‐prone short reads (Rho et al. 2010). Furthermore, applying screening thresholds tailored for environmental resistomics to account for divergent gene variants and fragmented scaffolds typical of complex aquatic metagenomes, aligning with screening strategies adopted in contemporary environmental resistome assessments (Ramalho et al. 2022; Bianco et al. 2022).

Fourteen AMR marker genes were identified across three of the four sampling points (SP0, SP1, and SP3), whereas SP2 yielded no detectable resistance determinants. The identified genes confer resistance to multiple antimicrobial classes, including β‐lactams, quinolones, sulfonamides, tetracyclines, macrolides, phenicols, and aminoglycosides. The sewage‐impacted reference point (SP0) exhibited the highest diversity and abundance of clinically relevant resistance genes. This profile included the β‐lactamase gene *bla*_ADC_ (assigned to *Acinetobacter* sp.), the macrolide resistance gene *mph*(E) (identified with 100% nucleotide identity to *Acinetobacter baumannii* ), two aminoglycoside phosphotransferase genes (*aph*(9)), and the tetracycline resistance gene *otr*(A) (Table 3).

**TABLE 3:** Characterization of antimicrobial resistance genes and microbial assignments from samples at Marambaia Island.

Site	Scaffold	Gene	Product	Resistance	Length	Identity (%)	Coverage (%)	Microbial assignment
SP1	26572	dfrA7	dfrA	Trimethoprim	225	87.25	17.54	*Escherichia coli*
	31980	tet(54)	Tetracycline destructase	Tetracycline	352	71.47	29.99	Uncultured bacterium
	29928	oqxB5	RND transporter permease subunit	Phenicol Quinolone	357	67.42	11.01	Acidobacterium
SP3	10710	catB_1	Chloramphenicol O‐acetyltransferase	Chloramphenicol	724	72.86	31.52	*Rhabdochlamydia* sp.
	123518	tet(51)	Tetracycline‐destructase	Tetracycline	364	94.00	25.86	*Rhabdochlamydia* sp.
	54566	vat(F)	Streptogramin A‐acetyl transferase	Dalfopristin Prostinamycon Virginiamycin M	391	71.67	34.98	Chlamydiota bacterium
	108404	rphD	Rifamycin‐inactivating phosphotransferase	Rifamycin	353	66.76	13.48	Patescibacteria group
	1654	mupA	mupirocin‐resistant isoleucine‐tRNA ligase MupA	Mupirocin	1157	73.71	5.63	Uncultured bacterium
SP0	12994	tet(56)	Tetracycline destructase	Tetracycline	533	79.56	38.46	*Legionella* spp.
	22774	aph(9)	Aminoglycoside O‐phosphotransferase	Spectinomycin	471	70.36	30.72	Uncultured bacterium
	49716	otr(A)	otr(A)	Doxycycline Tetracycline Minocycline	392	73.14	8.48	Spirochaetales
	59665	Mph(E)	Family macrolide 2	Macrolide	375	100.00	39.89	*Acinetobacter baumannii*
	9524	aph(9)	Phosphotransferase APH (9)‐IA	Spectinimycin	570	69.26	25.8	Uncultured Verrucomicrobiae
	21819	blaADC	Hydrolyzing C‐β‐lactamase ADC‐8	Cephalosporin	476	73.71	5.3	*Acinetobacter* sp.

In the freshwater source points, SP1 and SP3 each harbored distinct, fewer resistance determinants. SP1 contained the trimethoprim resistance gene *dfrA7* (assigned to *Escherichia coli* ) and the tetracycline destructase gene *tet*(54). SP3 yielded a broader array, including *tet*(51), *catB*_1_ (chloramphenicol resistance), *vat*(F) (streptogramin A acetyltransferase), *rphD* (rifamycin phosphotransferase), and *mupA* (mupirocin resistance). Tetracycline destructase genes *tet*(54), *tet*(51), and *tet*(56) were the most frequently detected resistance determinants across all three positive sites (Table 3). These genes are frequently reported in marine and estuarine environments, often carried by *Aeromonas* spp. and *Vibrio* spp., and may exhibit both host specificity and the capacity for horizontal transfer via plasmids (Ramalho et al. 2022; Suzuki et al. 2021).

This enrichment of clinically relevant determinants is consistent with the well‐established role of domestic sewage as a reservoir of antibiotic resistance genes, often associated with mobile genetic elements that facilitate their dissemination (Bengtsson‐Palme et al. 2018; Suzuki et al. 2021). The detection of clinically relevant AMR genes in a legally protected environmental area and distant from large urban centers is significant. AMR remains scarcely investigated in Brazilian environmental protection areas, with most studies focusing on urban and clinical settings. Ramalho et al. (2022) reported *bla*_TEM_ and *tet*(B) in urban water sources used for pre‐treatment intake in Brazil, alongside carbapenemase and quinolone residues in sewage samples.

The detection of AMR genes at Marambaia Island may be attributed to the island's geographic setting between the Atlantic Ocean and Sepetiba Bay, where marine spray, surface runoff, and tidal influence can introduce allochthonous genetic elements into coastal freshwater systems.

Similarly, Rout et al. (2023) detected *mph*(E) and *dfrA*, the same gene families identified in the present study, in water and sediments of the anthropogenically impacted Ganga and Yamuna rivers in India. The presence of these gene families at Marambaia Island demonstrates that even diffuse or intermittent anthropogenic pressures are sufficient to introduce clinically relevant resistance determinants into protected freshwater ecosystems.

Previous metagenomic studies in Rio de Janeiro state reinforce these observations. A plasmidome‐focused approach identified genes encoding metallo‐β‐lactamase enzymes in watersheds and drinking water samples (Bianco et al. 2022), and AMR genes were recently reported in polluted rivers of Ilha Grande Bay (Bacha et al. 2025). In the comparatively more preserved Marambaia Island, the detection of AMR genes may reflect not only local anthropogenic pressures, such as diffuse fecal contamination and recreational activities, but also the dispersal of resistance determinants from external sources mediated by marine vectors, underscoring the pervasive and interconnected nature of this environmental health challenge.

These findings reinforce the global concern that AMR has expanded far beyond clinical environments into environmental matrices, where it poses risks to both human and animal health and food and environmental safety (Sun et al. 2019). Future studies employing functional metagenomics, transcriptomics, or culture‐based phenotypic assays are warranted to validate the activity and public health relevance of the resistance determinants detected here and to quantify the actual exposure risk associated with the use of these freshwater sources.

### 3.4 Inference of Ecological Patterns Consistent With Anthropogenic Impacts on Microbial Communities of Water Sources of Marambaia Island

The taxonomic and diversity analyses demonstrated that microbial community structure varies among the sampling points at Marambaia Island, with sites differing in both composition and richness. To determine whether these differences correspond to measurable environmental differences and can be quantitatively linked to anthropogenic pressure, a suite of integrative analyses was conducted, combining physicochemical parameters, bacterial indicator taxa, and a customized Anthropogenic Impact Index (AII) calculated for each sampling point, defined as the ratio between the cumulative relative abundance of contamination‐associated bacterial markers and the total microbial community abundance.

The use of microbial community profiles as indicators of anthropogenic disturbance is also supported by metagenomic investigations in other Brazilian coastal ecosystems. In the Baía de Todos os Santos, in the Northeast of Brazil, Carvalho et al. (2024) identified distinct microbial and functional signatures between preserved and anthropogenically disturbed mangroves, suggesting that metagenomic profiles can serve as indicators of environmental degradation.

To assess human impact, contamination markers were selected to encompass enteric bacteria, opportunistic pathogens, and fecal indicators (*Escherichia*, *Enterococcus*, *Legionella*, *Pseudomonas*, *Acinetobacter*, *Vibrio*, *Clostridium*, *Bacteroides*, *Streptococcus*, and *Campylobacter*), supported by McLellan et al. (2024), who highlighted the global utility of bacterial molecular markers for detecting aquatic fecal contamination. In contrast, environmental markers included genera characteristic of minimally impacted freshwater and soil systems, such as *Bradyrhizobium* and *Streptomyces*.

**FIGURE 5:**
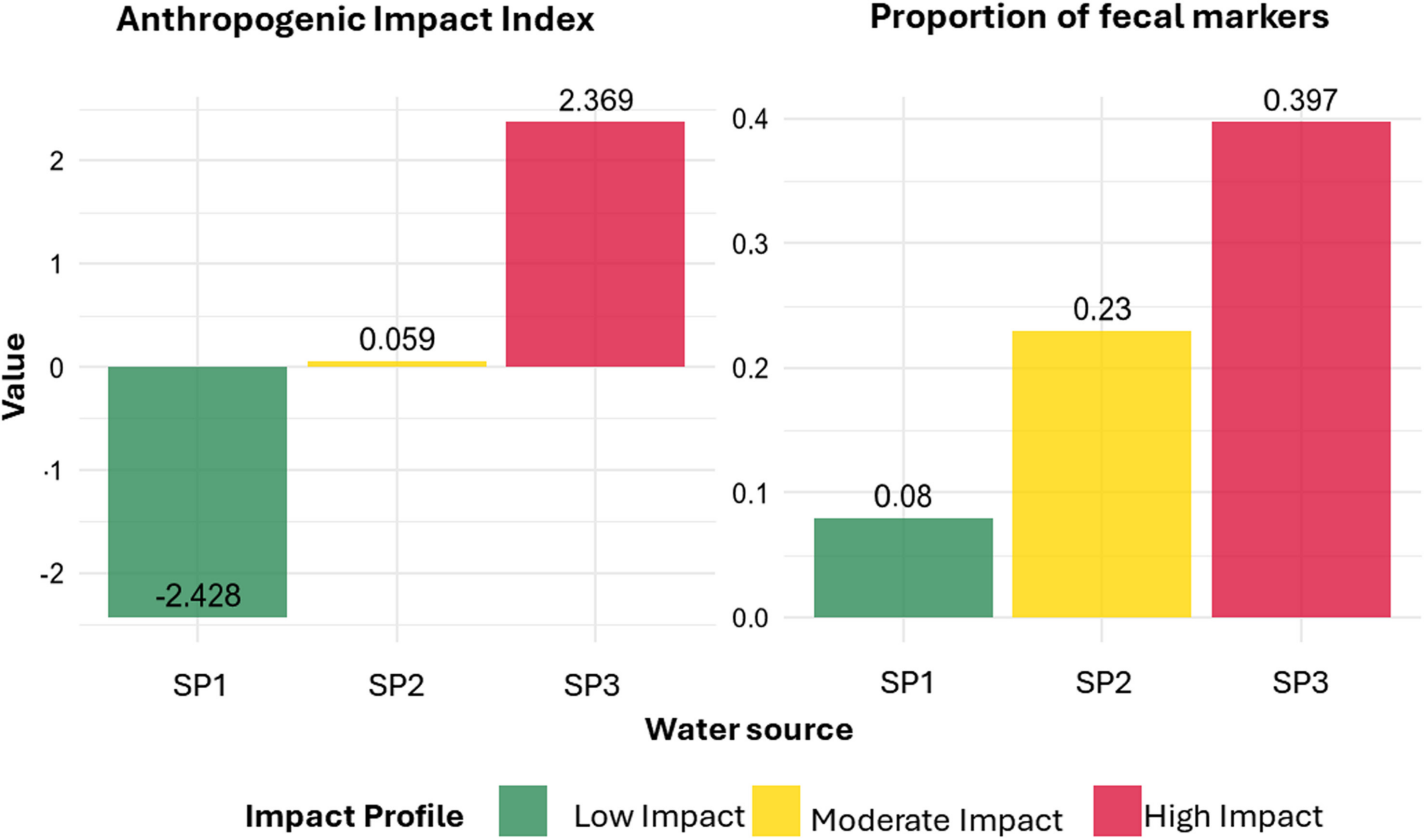
Anthropogenic impact estimation by Anthropogenic Impact Index and proportion of fecal markers in the water sources of Marambaia Island.

Distinct levels of anthropogenic impact were observed across the sampling sites, as reflected by the AII and corroborated by the proportion of fecal‐associated markers at each location (Figure 5). SP1 exhibited the lowest AII and fecal marker proportion, consistent with its high species richness and even community structure. SP3, conversely, displayed the highest values for both metrics. SP2 occupied an intermediate distinct position.

Redundancy analysis (RDA) was employed to quantify the extent to which physicochemical variables explain the variation in microbial community composition across sites. The environmental variables included in the model were salinity, conductivity, turbidity, pH, total dissolved solids (TDS), and dissolved oxygen (DO). The AII was incorporated as a supplementary variable.

The Redundancy analysis (RDA) revealed that 88.1% of the variation in microbial communities is explained by the environmental variables, with the RDA1 axis (69.8% of the variance) represented by the parameters conductivity, salinity and turbidity, which have a high correlation with genera associated with impacted environments, such as *Legionella*, *Pseudoalteromonas* and Enterobacteria, in addition to the Anthropogenic Impacts Index (AII) as a supplementary variable.

**FIGURE 6:**
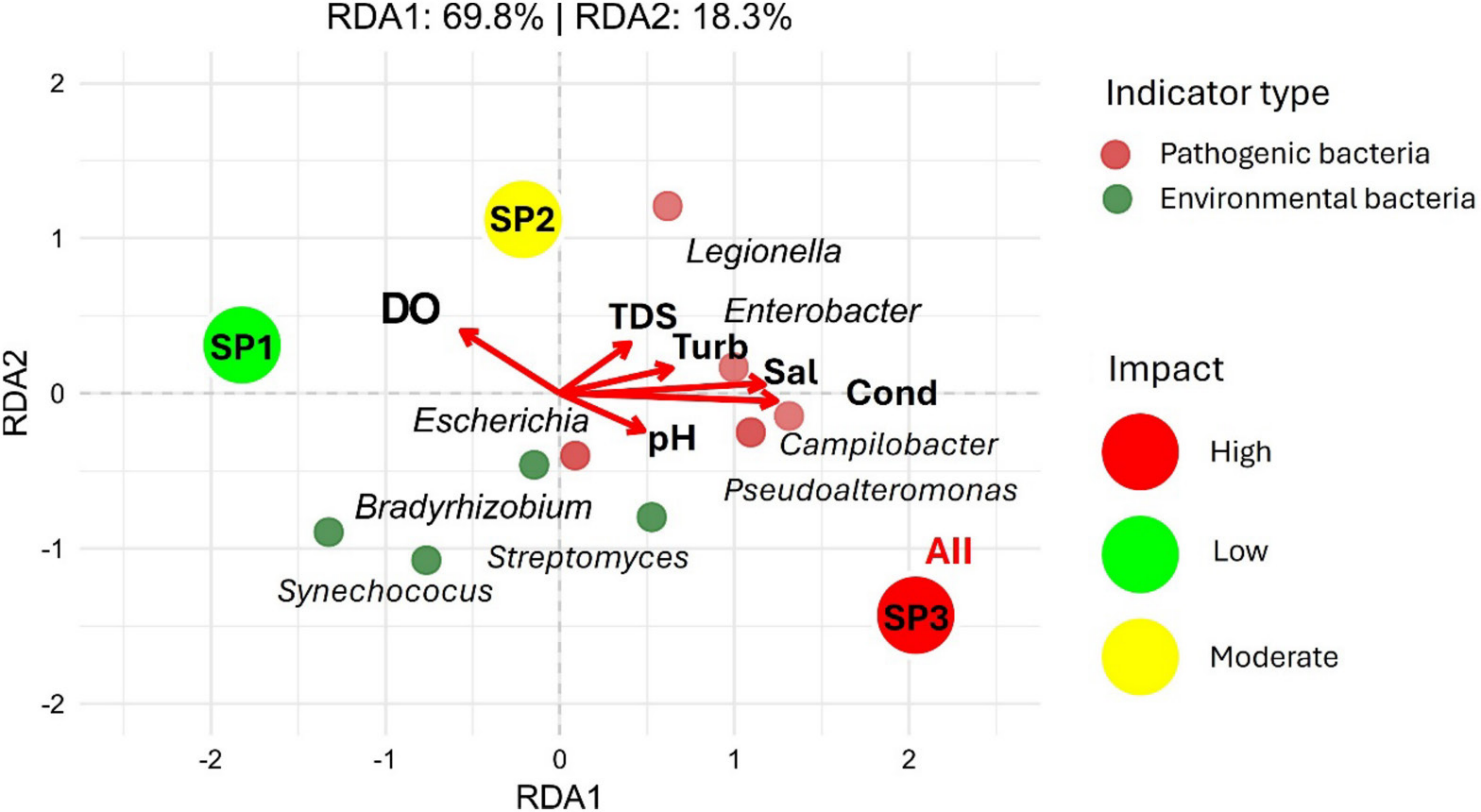
Variation of the microbial community explained by the physicochemical characteristics in the study water sources according to redundancy analysis.

Although on the RDA2 axis (18.3% of the variance) the genera *Bradyrhizobium* and *Streptomyces* were associated with conditions of low anthropogenic impact, such as high levels of DO in SP1. The presence of the genus *Escherichia* in the three regions points to the diffuse anthropogenic influence in the three water sources, regardless of the environmental factor (ANOVA by permutation—999 permutations; F = 8.45, *p* = 0.001; RDA1: F = 12.67, *p* = 0.002; RDA2: F = 3.24, *p* = 0.048) as shown in Figure 6. Metagenomic and microbial evaluations conducted across coastal and estuarine ecosystems in Southeastern Brazil (Taketani et al. 2018) revealed that anthropogenic nutrient loading and untreated domestic effluents act as dominant environmental drivers that restructure bacterial assemblies, resulting in a marked shift from native aquatic taxa to human‐associated fecal bioindicators and potential pathogens.

The variation in anthropogenic impact observed across the evaluated water sources is corroborated by studies employing similar multivariate approaches to link environmental variables to microbial community structure (Köchling et al. 2017; Rout et al. 2023).

The strong correlation observed between the RDA1 axis and parameters such as conductivity, salinity, and turbidity, coupled with the differences in fecal markers (ranging from 8% at baseline sites to 39.7% at more impacted locations) and the Anthropogenic Impact Index (AII), aligns with the findings of Liu et al. (2023) in the Yellow River Delta when the authors identified that water depth and Na^+^ and Ca^2+^ content were the most significant environmental factors affecting the microbial community in areas subjected to intense anthropogenic activity, particularly due to the discharge of industrial and domestic effluents.

Furthermore, findings from Brazilian studies involving freshwater microcosms contaminated with hydrocarbons (Jurelevicius et al. 2013), in which RDA revealed the selection of specific genera (*Acinetobacter* and *Cloacibacterium*) in response to different pollutant types, identified associations between genera (*Legionella, Pseudoalteromonas, Enterobacteriaceae*) and the anthropogenic impact profile, suggesting that the microbial community responds predictably to environmental stressors.

Similar to the observations in the present study, Lima (2015) demonstrated through RDA applied to the Tietê River basin in São Paulo State that physicochemical variables and the geographic location of sampling points explained the distribution of bacterial genera along the water quality gradient, corroborating the structuring role of environmental parameters on the microbial community in Brazilian freshwater ecosystems, as observed in the transition from genera such as *Bradyrhizobium* and *Streptomyces*, associated with low‐impact conditions and high DO levels at SP1, to *Legionella*, *Pseudoalteromonas*, and *Enterobacteriaceae*, correlated with the impact levels at SP2 and SP3.

The elevated AII and stronger human‐associated microbial signature observed at SP3 are also consistent with its closer spatial proximity to the densely populated area, relative to the greater distance separating this area from SP2. This spatial pattern suggests that hydrological and physical connectivity to anthropogenic sources, rather than population density per se, may be a key driver of localized contamination signatures. Such a distance‐dependent pattern is consistent with the diffuse, gradient‐like nature of contamination typically observed in freshwater systems influenced by runoff, subsurface flow, and salt spray deposition in coastal settings.

This reinforces the interpretation that the low AII values observed at the most densely populated site do not necessarily reflect an absence of anthropogenic pressure, but rather a spatial decoupling between the source of pressure and its microbial manifestation downstream or laterally. Taken together, these findings suggest that informal protective practices directed at this water source may play a determining role in maintaining microbial balance and preserving water quality at SP1, despite its proximity to a densely populated area and the absence of formal prior treatment.

Overall, the integration of microbial community composition, AMR profiles, and anthropogenic signatures revealed distinct patterns of human‐associated influence across the studied freshwater sources. These patterns are consistent with previous studies showing that anthropogenic pressures and local environmental conditions can restructure freshwater microbial communities and favor the persistence or enrichment of specific microbial groups associated with contamination (Taketani et al. 2018; Rout et al. 2023). Taken together, our findings demonstrate that the integration of taxonomic, resistance, environmental, and anthropogenic indicators provides a more comprehensive assessment of human‐associated microbial pressures in freshwater ecosystems.

## 4 Conclusions

This study aimed to characterize microbial community composition and dynamics across water sources within a protected coastal area, assessing patterns of anthropogenic impact, environmental drivers of microbial structure, and the occurrence of AMR genes. The findings indicate that anthropogenic pressures shape microbial community composition and are associated with the enrichment of clinically relevant AMR determinants across coastal freshwater sources on Marambaia Island.

Sites under greater anthropogenic influence consistently displayed reduced microbial richness, a higher number of detected AMR genes, and enrichment of bacterial taxa associated with human activity and fecal contamination, with the Anthropogenic Impact Index (AII) supporting a clear human‐associated microbial signature at these sites. Particularly, this pattern was not simply a function of population density. The site with the highest degree of human occupation displayed a more balanced microbial profile, combining substantial taxonomic diversity, a mix of environmental and potentially pathogenic bacterial genera, and comparatively low AII values, with RDA indicating limited influence of environmental contaminants at this location.

This may reflect some degree of care directed at this water source, despite the apparent absence of formal prior treatment, suggesting that factors such as protective practices, water use patterns, and hydrological connectivity can influence microbial disturbance more than occupation density alone. Particularly, AMR genes were detected across nearly all sampling sites, including both sewage‐impacted and freshwater sources, with the exception of the most distant and least populated site, reinforcing that resistance dissemination is not confined to urban or clinical settings and may depend on site‐specific environmental and infrastructural factors that remain insufficiently understood.

Although traditional physicochemical and microbiological approaches remain essential for regulatory compliance, high‐resolution metagenomic surveillance offers a complementary perspective, revealing genetic risks that conventional indicators may not capture. By coupling an Anthropogenic Impact Index with RDA, this study provides a replicable framework for assessing diffuse contamination in protected coastal areas, sensitive to context‐specific drivers of human impact beyond occupation density alone.

These results should be interpreted considering certain limitations, including the number of sampling sites and time points, which may not fully capture seasonal or hydrological variability, and the taxonomic and functional resolution inherent to the metagenomic and bioinformatic approaches employed. Additionally, informal site‐specific protective practices that may influence water source conditions were not systematically documented in this study, and their potential role in shaping the microbial patterns observed, particularly at the site with the highest population density, warrants further investigation.

Given the vulnerability of freshwater sources in protected yet human‐accessible areas, these findings underscore the need for integrated management strategies combining traditional monitoring with metagenomic investigation, supported by adequate sanitation infrastructure and targeted environmental health education to mitigate contamination pathways and safeguard both ecosystem resilience and public health.

## Author Contributions

**Bruna Barbosa de Paula:** investigation, writing – original draft, methodology, formal analysis, validation. **Marize Pereira Miagostovich:** conceptualization, funding acquisition, writing – review and editing, supervision. **Camille Ferreira Mannarino:** funding acquisition, writing – review and editing. **André Vinicius Costa Ribeiro:** writing – review and editing, methodology. **Natália Maria Lanzarini:** methodology, writing – review and editing. **Carla Santos de Oliveira:** methodology, writing – review and editing. **Shênia Patrícia Corrêa Novo:** writing – review and editing, supervision, project administration.

## Funding

This work was supported by Brazilian National Health Foundation ‐ FUNASA (Project 25388.010001/2018‐23), Conselho de Desenvolvimento Científico e Tecnológico (CNPq 0406080/2021–CNPq 305737/2023‐6; CNPq PROEP/IOC441653/2024‐3) and Fundação de Amparo à Pesquisa do Rio de Janeiro (FAPERJ E26/202.266/2024).

## Ethics Statement

The authors have nothing to report.

## Conflicts of Interest

The authors declare no conflicts of interest.

## Data Availability

Raw reads are publicly available in the Sequence Read Archive (SRA) (NCBI—https://www.ncbi.nlm.nih.gov/sra) individually with accession numbers (SAMN48402021, SAMN48402022, SAMN48402023, SAMN48402024, SAMN48402025) under BioProject accession number (PRJNA1260703).

## Appendix A Physicochemical Parameters of Freshwater Sampling Sites

**TABLE A1:** Physicochemical parameters monitored monthly from January to December 2022 at freshwater sampling sites on Marambaia Island, Rio de Janeiro, Brazil. Adapted from de Paula et al. (2024).

Parameters	Region	Median	Minimum	Maximum
pH	SP1	6.47	5.38	8.52
	SP2	6.54	5.75	7.90
	SP3	6.85	5.91	8.24
Conductivity (mS cm^−1^)	SP1	0.079	0.061	0.118
	SP2	0.095	0.083	0.105
	SP3	0.141	0.102	0.173
Turbidity (NTU)	SP1	3.65	0.00	15.50
	SP2	6.42	0.00	44.20
	SP3	7.39	0.90	17.40
Dissolved oxygen (mg L^−1^)	SP1	8.75	6.33	11.07
	SP2	6.34	5.15	10.50
	SP3	7.13	3.05	10.85
Temperature (°C)	SP1	20.28	10.17	27.34
	SP2	22.16	17.76	30.50
	SP3	21.86	16.58	31.24
Salinity (ppt)	SP1	0.03	0.03	0.05
	SP2	0.04	0.04	0.05
	SP3	0.06	0.05	0.08
Reduction potential (ORPmV)	SP1	175.25	79	276
	SP2	188.00	137	240
	SP3	168.00	74	249
Total dissolved solids (g L^−1^)	SP1	0.051	0.040	0.076
	SP2	0.060	0.040	0.086
	SP3	0.066	0.066	0.120
